# Callosal connections of dorsal versus ventral premotor areas in the macaque monkey: a multiple retrograde tracing study

**DOI:** 10.1186/1471-2202-6-67

**Published:** 2005-11-25

**Authors:** Driss Boussaoud, Judith Tanné-Gariépy, Thierry Wannier, Eric M Rouiller

**Affiliations:** 1 Institut de Neurosciences Cognitives de la Méditerranée, INCM, UMR 6193, CNRS, Université de la Méditerranée, 31 Ch. Joseph Aiguier, 13402 Marseille Cedex 20, France; 2 Unit of Physiology and Program in Neurosciences, Department of Medicine, University of Fribourg, Rue du Musée 5, CH-1700 Fribourg, Switzerland; 3 Brain Research Institute, Dept. Neuromorphology, University and ETH Zurich, Winterthurerstr. 190, CH-8057 Zürich, Switzerland

## Abstract

**Background:**

The lateral premotor cortex plays a crucial role in visually guided limb movements. It is divided into two main regions, the dorsal (PMd) and ventral (PMv) areas, which are in turn subdivided into functionally and anatomically distinct rostral (PMd-r and PMv-r) and caudal (PMd-c and PMv-c) sub-regions. We analyzed the callosal inputs to these premotor subdivisions following 23 injections of retrograde tracers in eight macaque monkeys. In each monkey, 2–4 distinct tracers were injected in different areas allowing direct comparisons of callosal connectivity in the same brain.

**Results:**

Based on large injections covering the entire extent of the corresponding PM area, we found that each area is strongly connected with its counterpart in the opposite hemisphere. Callosal connectivity with the other premotor areas, the primary motor cortex, prefrontal cortex and somatosensory cortex varied from one area to another. The most extensive callosal inputs terminate in PMd-r and PMd-c, with PMd-r strongly connected with prefrontal cortex. Callosal inputs to PMv-c are more extensive than those to PMv-r, whose connections are restricted to its counterpart area. Quantitative analysis of labelled cells confirms these general findings, and allows an assessment of the relative strength of callosal inputs.

**Conclusion:**

PMd-r and PMv-r receive their strongest callosal inputs from their respective counterpart areas, whereas PMd-c and PMv-c receive strong inputs from heterotopic areas as well (namely from PMd-r and PMv-r, respectively). Finally, PMd-r stands out as the lateral premotor area with the strongest inputs from the prefrontal cortex, and only the PMd-c and PMv-c receive weak callosal inputs from M1.

## Background

**Figure 1:**
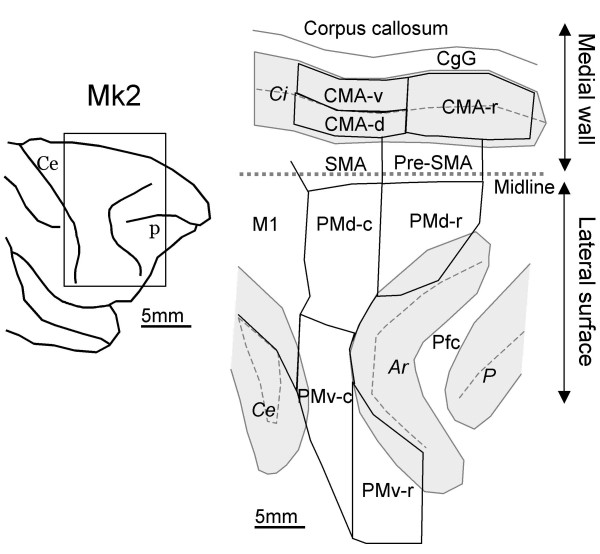
Premotor areas represented on a two-dimensional map of the cortex. On the left, surface view of the anterior part of the right hemisphere. The rectangle indicates the cortical region flattened and shown on the right. On the 2-D map, sulci are represented by shaded zones, the dashed lines indicate the fundus of the sluci. The premotor subdivisions are defined on the basis of SMI-32 staining (see text). Abbreviations: Ar, arcuate sulcus; Ce, central sulcus; CgG, cingulate gyrus; Ci, cingulate sulcus; CMA-d, r and v, dorsal rostral and ventral parts of the cingulate motor area, respectively; P = sulcus principalis; M1, primary motor cortex; PMd-c, r, caudal and rostral parts of the dorsal premotor cortex; PMv-c, r, caudal and rostral parts of the ventral premotor cortex; pre-SMA, rostral part of the SMA; SMA-proper, caudal part of the SMA.

The motor cortex of macaques is divided into four main regions: the primary motor cortex (M1), the premotor cortex (PM), the supplementary motor area (SMA) and the cingulate motor area (CMA). These regions have been subdivided further into distinct areas on the basis of anatomical and functional criteria. In the PM region, the dorsal (PMd) and ventral (PMv) areas have been distinguished on anatomical, histochemical and neurophysiological ground [1-6]. More recently, PMd and PMv have been proposed to contain distinct functional areas along the rostro-caudal axis, referred to as PMd-r, PMd-c, PMv-r and PMv-c [5,7-9]. They correspond roughly to areas F7, F2, F5 and F4, respectively, in the nomenclature of Matelli and his co-workers [10-14]. Similarly, SMA has been subdivided into a rostral part (pre-SMA) and a caudal part (SMA-proper) [15], also referred to as F6 and F3, respectively [10,11,13,14,16]. Finally, three areas have been identified within the CMA on the basis of corticospinal projections [17]: a rostral area (CMA-r) and two caudal areas, one dorsal (CMA-d) and one ventral (CMA-v). These multiple subdivisions are illustrated in Figure 1.

The ipsilateral connections of these motor cortical areas with the other cortical areas have been extensively studied since many years, with renewed interest in recent years especially with respect to the posterior parietal cortex [1,7,10-12,18-60]. By contrast, callosal connections of most premotor areas have attracted less attention, despite their importance for understanding inter-hemispheric exchange of information necessary for coordinated actions of the two sides of the body [61]. It is thus of interest to know how each premotor area connects with the opposite hemisphere in terms of topography and strength of the connections. Previous studies have described the callosal connectivity of M1 and SMA-proper [31,61,62]. They have shown that the hand area of M1 receives a minor callosal input from its counterpart in the other hemisphere, whereas the hand area in SMA-proper is more densely interconnected with the other hemisphere. More recently, Liu et al. [63] have contrasted the callosal connections of SMA-proper and pre-SMA and found that the two areas share common callosal inputs but the strength of the connections differs, with pre-SMA more heavily connected with the opposite hemisphere.

Callosal connectivity of the other premotor areas has been less investigated. Only one recent study [64] has described the callosal connections of the rostral and caudal dorsal premotor areas (PMd-r and PMd-c, corresponding to the areas F7 and F2, respectively), whereas those of ventral premotor and cingulate motor areas are still lacking. We performed an extensive multiple tracing investigation of callosal connections of the lateral premotor areas, with emphasis on the distinction between dorsal (PMd) and ventral (PMv) sectors as well as the comparison between their rostral and caudal divisions. We compared data obtained from two groups of animals. In the first group, large injections of 3–4 tracers were performed in each animal (n = 3) filling in most, if not the whole, extent of the PM sub-areas. In a second group of animals (n = 5), we performed smaller injections in the PM sub-areas for comparison with other studies. The first group of animals was used to describe a fairly exhaustive picture of the origin of the callosal projection to the four sub-areas of PM, including the issue of overlap/segregation of the different projections, whereas more precise topographic aspects are described based on the second group of animals.

## Results

### Injection sites

**Figure 2:**
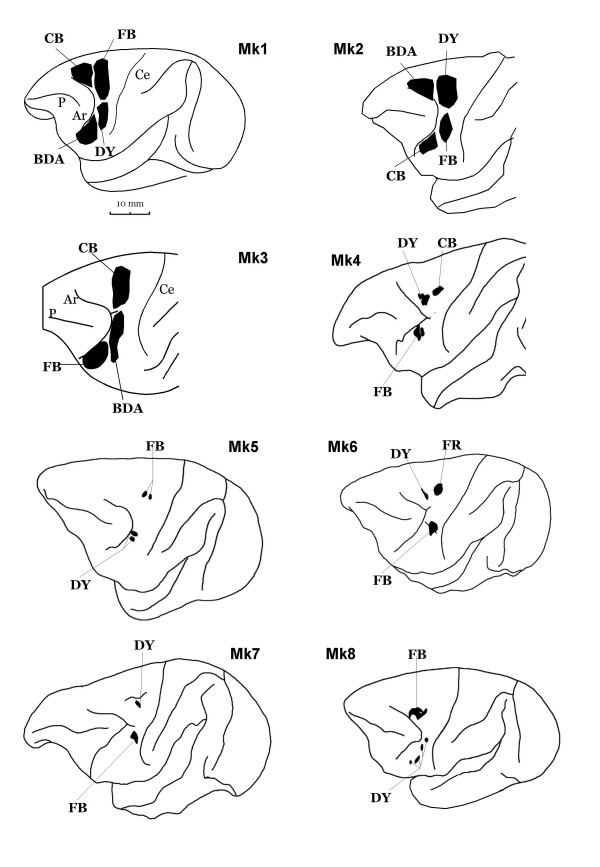
Reconstruction of the injection sites on a lateral view of the left hemisphere of the 8 monkeys included in the present study. Tracers: biotinylated dextran amine (BDA), diamidino yellow (DY), fast blue (FB), fluoro ruby (FR), cholera-toxin B subunit (CB). For other abbreviations, see Fig. 1.

The locations of the injection sites were confirmed on histological criteria. Figure 2 shows the reconstruction of each injection site on surface views of the brain hemisphere. Each monkey received 2–4 distinct tracers injected in different PM areas. As the figure shows, the injection sites varied in size and location within each PM sub-area and, sometimes, encroached on an adjacent area (see also Table I). A particular protocol was conducted in Mks 1–3 in order to obtain large injection sites covering most of the injected PM sub-area. Examples of such large injection sites are shown on photomicrographs (Fig. 4), following injections of BDA, DY, FB and CB. The injections were performed in such a way (usually at 2 depths along each penetration) to form a cylinder covering all cortical layers, from the surface down to the limit between the grey and the white matter.

**Figure 3:**
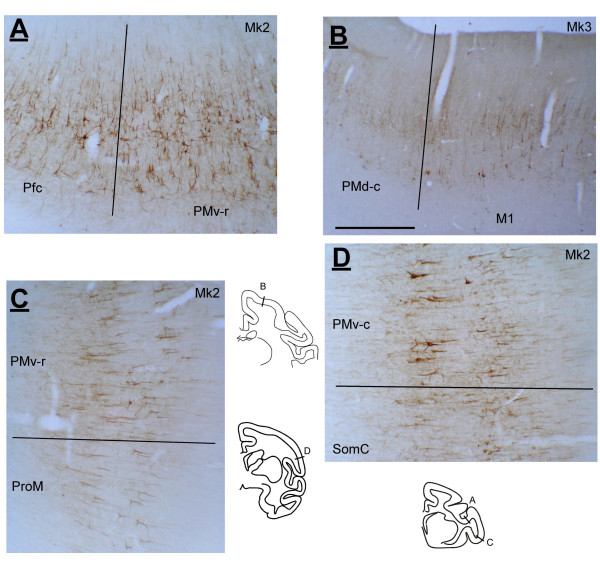
Photomicrographs showing SMI-32 staining observed in Mk2 or Mk3 illustrating transition zones between the prefrontal cortex (Pfc) and PMv-r (panel A), between PMd-c and M1 (panel B), between PMv-r and ProM (panel C) and PMv-c and SomC (panel D). See list of abbreviations. Scale bar = 1 mm.

**Table 1:** List of tracers injected in PM in Monkeys Mk1 – Mk8, with indications on the total volume injected for each tracer, the number of penetrations and sites of infusions.

	**PMd-r**	**PMd-c**	**PMv-r**	**PMv-c**
**Mk1**	*CB*	*FB*	*BDA*	*DY*
	x (2.7 μl,5,9)	x (7 μl,7,7)	x (9 μl,6,9)	x (16 μl,8,10)
**Mk2**	*BDA*	*DY*	*CB*	*FB*
	x (20 μl,10,20)	x (12 μl,6,12)	x (3.9 μl,7,13)	x (12 μl,6,12)
**Mk3**		*CB*	*FB*	*BDA*
		x (2.5 μl,3,5)	x (9 μl,7,9)	x (6 μl,6,6)
**Mk4**	*DY*	*CB*	*FB*	
	x (0.4 μl,2,4)	x (7.5 μl,2,4)	x (0.8 μl,2,4)	
**Mk5**		*FB*	*DY*	
		W, E (0.4 μl,2,4)	D, W (0.6 μl,2,4)	
		70–80 μA	50 μA	
**Mk6**	*DY*	*FR*		*FB (M1)*
	NE (0.4 μl,2,4)	NE (1 μl,2,4)		F (0.8 μl,2,4)
**Mk7**		*DY*		*FB*
		E, S (0.6 μl,2,4)		NE (8 μl,2,4)
		75 μA		
**Mk8**	*FB*		*DY*	
	Eyes (0.8 μl,4,8)		F (0.8 μl,4,4)	
	10–20 μA		12–80 μA	

x = No ICMS performed. NE: non-excitable site with ICMS.

Conventions for ICMS: D = Digits; E = Elbow; F = Face; S = Shoulder; W = wrist.

Tracers: biotinylated dextran amine (BDA), diamidino yellow (DY), fast blue (FB), fluoro ruby (FR), cholera-toxin B subunit (CB). Arrows means that the injection site encroaches the adjacent area pointed by the arrow. (Ml) indicates that the injection site in PMd-c or PMv-c encroaches Ml.

Below each tracer, the three numbers between parentheses give the total volume injected, the number of syringe penetrations and the total number of sites where the tracer was infused.

**Figure 4:**
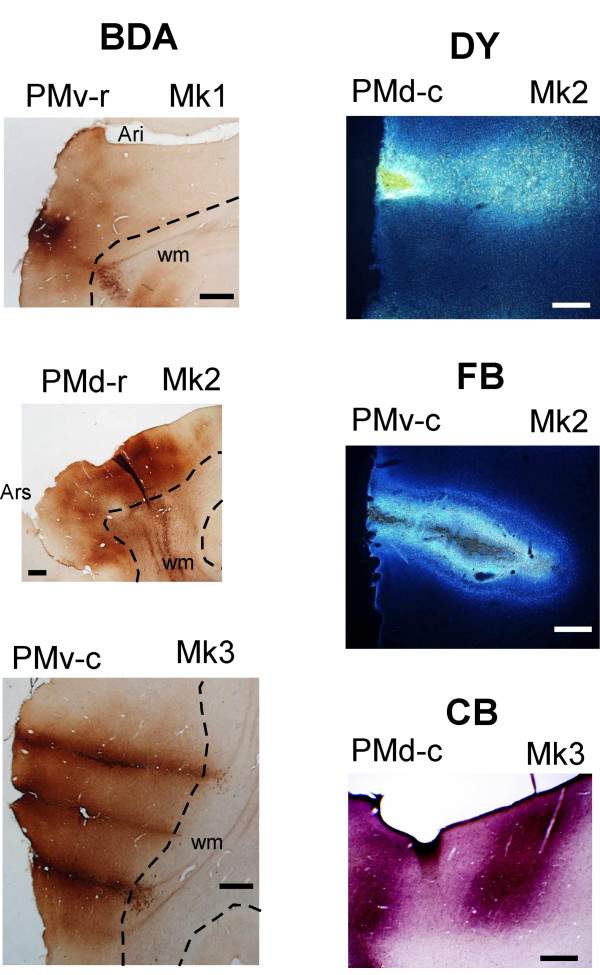
Photomicrographs of typical injection sites for BDA (left column) and for DY, FB and CB (from top to bottom in the right column). Scale bar = 1 mm.

**Figure 5:**
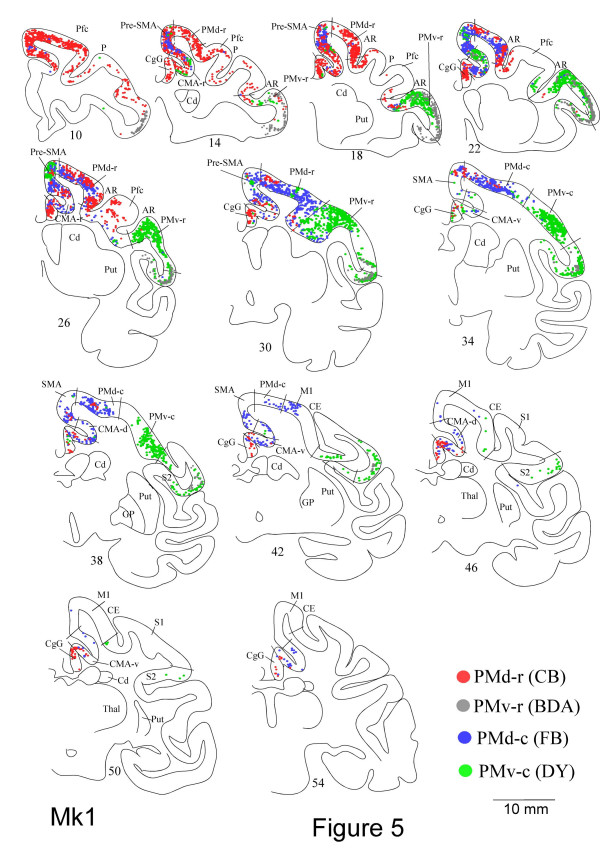
Frontal sections of the right hemisphere of Mk1, arranged from rostral to caudal with increasing ID# (10 to 54), showing the distribution of retrogradely labelled neurons as a result of tracers injections in the opposite PMd-r (red dots), PMv-r (grey dots), PMd-c (blue dots) and PMv-c (green dots). The tracers used are indicated in the bottom right. Tracers: biotinylated dextran amine (BDA), diamidino yellow (DY), fast blue (FB), cholera-toxin B subunit (CB). See list of abbreviations.

**Figure 6:**
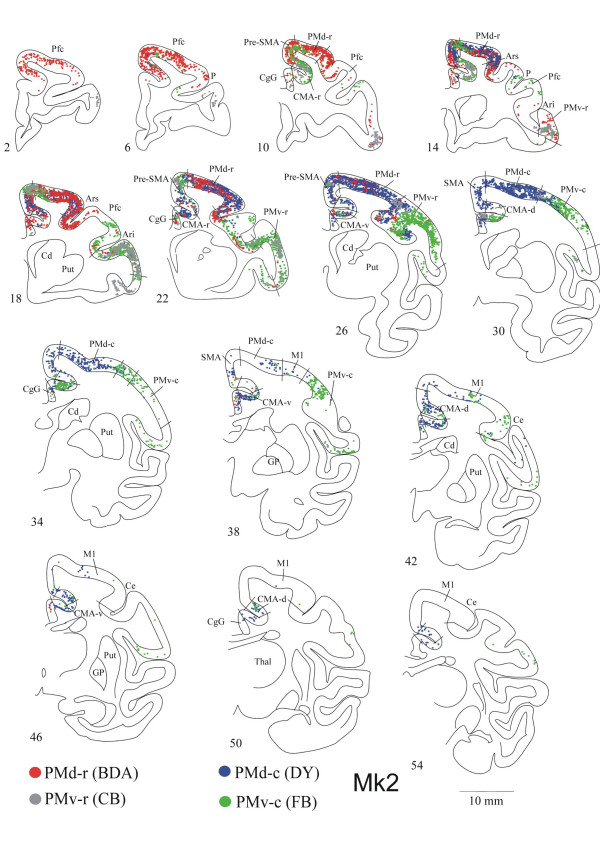
Frontal sections of the right hemisphere of Mk2, showing the distribution of retrogradely labelled neurons as a result of tracers injections in the opposite PMd-r (red dots), PMv-r (grey dots), PMd-c (blue dots) and PMv-c (green dots). Same conventions as in Figure 5.

In the following sections, we will describe the callosal labelling based on representative examples of the results obtained in MK1 and MK2 (Fig. 5 and 6). Individual variability is illustrated in figure 8 for 11 cases, and additional tables [Additional file 1] and a figure [Additional file 2] are presented as *Supplementary material*. In Figures 5 and 6, we have chosen to superimpose the labelling from 4 different tracers for comparison reasons.

### Injections in PMd-r

**Figure 7:**
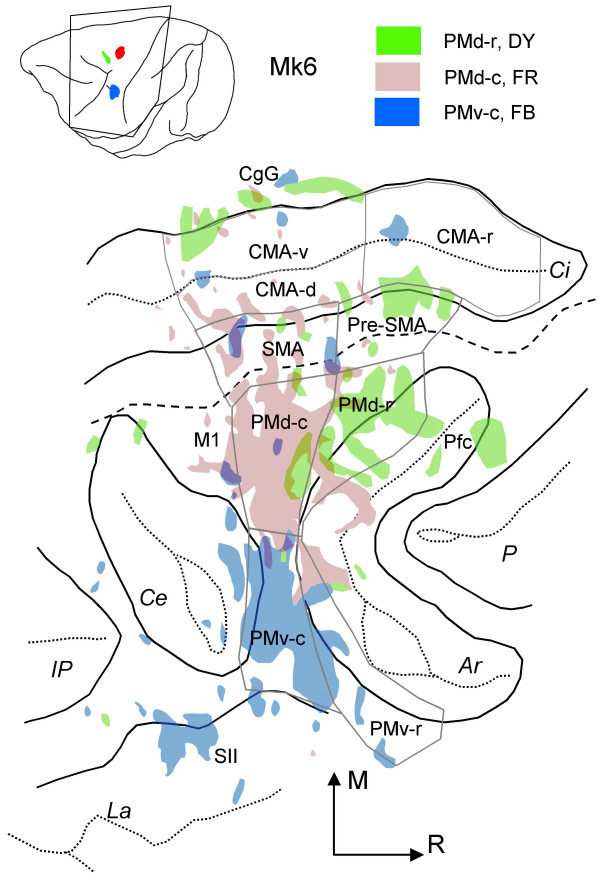
Distribution of labelling after 3 injections in Mk6 illustrated on a 2-D map of the frontal cortex. Same abbreviations and conventions as in Fig. 1.

Five injections were made into PMd-r (Table I). Figures 5 and 6 (red dots) illustrate the distribution of retrogradely labelled cells in the hemisphere contralateral to the injection site following large injections into PMd-r of two monkeys. As the figures show, PMd-r receives it main callosal projections from the premotor areas anterior to the level of the genu of the arcuate sulcus, and from prefrontal cortex. Within this region, labelling was assigned to PMd-r, Pfc dorsal to the principal sulcus extending to the cingulate sulcus, pre-SMA and rostral cingulate cortex (CMA-r). In the cortex located laterally or caudally to the level of the genu of the arcuate sulcus, labelling was sparse or limited to small patches (Pfc ventral to the principal sulcus, PMv-r, PMd-c, CMA-v). This general pattern of transcallosal labelling was consistent with the data derived from a smaller injection of DY in PMd-r in Mk 6 (see Fig. 7, green patches), although the labelling was less extensive.

**Figure 8:**
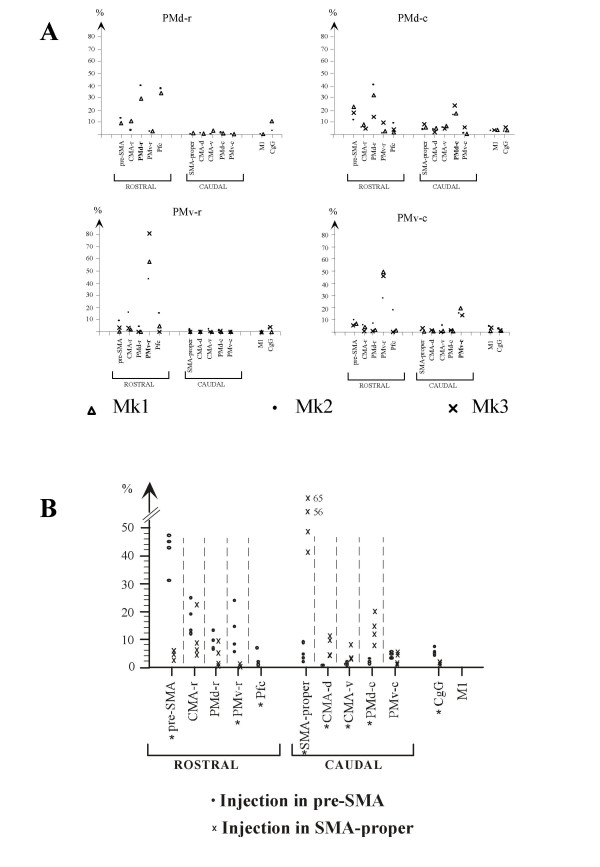
A: quantitative data giving the percent distribution of callosal neurons observed in different cortical areas as a result of tracer injections made in PMd-r (top left plot), PMd-c (top right plot), PMv-r (bottom left plot) and PMv-c (bottom right plot). The percent values are given by different symbols for each of the three individual monkeys included in the quantitative analysis (Mk1, Mk2 and Mk3). For a given monkey, the sum of the percent values is 100%. B: for comparison, same data, but for the distribution of callosal neurons projecting to pre-SMA and SMA-proper for other monkeys (taken from [63]).

### Injections in PMd-c

Seven injections were made in PMd-c in 7 animals (Table I). Figures 5 and 6 illustrate the distribution of labelling on coronal sections (blue dots), and figure 7 shows the data for monkey Mk6 on a 2-D map of the cortex. As after injections in PMd-r, injections in PMd-c yielded extensive labelling in the dorso-medial frontal cortex of the contralateral hemisphere. The main difference is that here, the labelling was relatively more caudal than following injections in PMd-r (compare red and blue dots). Analysis of the distribution of labelled cells in relation with areal borders shows that the strongest labelling was located in PMd-c, PMd-r and pre-SMA. Moderate or weak labelling was also found in the cingulate motor areas (CMA-r, CMA-v and CMA-d), SMA-proper and M1. The general pattern of labelling was the same in Mk3 where large injections were made, except that the labelling was more predominant in PMd-c than in PMd-r (see Fig. 8A and supplementary material). The results following a small injection in PMd-c are shown in Fig. 7, and they confirm the main observations made on the basis of large injection. The main difference is that the labelling was less extensive in rostral PMd-r following a small injection.

### Injections in PMv-r

Six injections were made in PMv-r of 6 different animals (Table I). The key finding is that following these injections, most labelled cells in the contralateral hemisphere were found in the cortex located just behind the inferior arcuate sulcus, anterior to the level of the genu (sections 14–22 in Fig. 5 and 6, grey dots), which corresponds to the counterpart area PMv-r. As one moves anteriorly or posteriorly, the dense labelling in PMv-r moves ventrally, forming a long stripe within the bank of the lateral sulcus (Fig. 5 and 6; see also supplementary figure). At its caudal aspect, this labelling is probably in area S2. Additional labelling was found in pre-SMA, CMA-r and ventral Pfc (Fig. 6). Note that labelling was observed in dorsal premotor areas (Fig. 6, section 22), but this projection was not confirmed in the other cases with similar injections. Finally, there was no labelling in PMv-c, i.e. behind the genu.

### Injections in PMv-c

Five injections were made in PMv-c (Table I). These injections gave rise to strong labelling in the contralateral frontal areas, with the core of labelling in PMv-c and PMv-r in all cases. Figures 5 and 6 illustrate two representative examples (green dots). As in the cases with injections in PMv-r, callosal labelling following injections in PMv-c is located mainly lateral to the genu of the arcuate sulcus and in mesial cortex. The most extensive labelling was found in the ventral premotor region (including both PMv-r and PMv-c), where it spanned the cortex caudal and anterior to the level of the genu of the arcuate sulcus (sections 18–38 in Fig. 5 and 6). Weak labelling was found consistently in pre-SMA, CMA-r and M1, and in some cases in PMd-r, SMA-proper, CMA-v and CMA-d and PMd-c (see Fig. 8).

### Comparison between PMd and PMv

The present study allowed a direct comparison between the callosal connections of the four premotor areas investigated. Comparison can be made directly on coronal sections in figures 5 and 6 (two monkeys with large injections of 4 tracers each) and on a 2D map of the cortex in figure 7, in monkey Mk6 where we made small injections of 3 tracers (see also Fig. 8).

It appears that, at a gross level, callosal projections to dorsal and ventral premotor sectors are organized along both the rostro-caudal and the medio-lateral axes (Fig. 5 and 6). Along the rostro-caudal axis, injections in rostral sectors (PMd-r and PMv-r) tend to yield stronger labelling in rostral frontal areas of the opposite hemisphere, i.e. anterior to the level of the genu of the arcuate sulcus (see for example Fig. 6; red and grey dots). Similarly, large injections in the caudal sectors (PMd-c and PMv-c) resulted in strong callosal labelling in caudal frontal areas (blue and green dots), with however, important labelling in rostral regions overlapping with the projections to rostral sectors. This might be due to the large size of the injection sites, as small injections into PMd-c and PMv-c (Fig. 7) led to less overlap. A similar pattern of labelling is also observed following injections into PMv-c (green dots in Fig. 5 and 6). Figure 8A shows the percentage of cells in different areas, organized a rostral and a caudal group.

Superimposed to this trend, the organisation of callosal projections along the medio-lateral axis is even more striking. Figures 5 and 6 show that callosal inputs to PMd arise almost exclusively from the dorso-medial regions (red and blue dots), whereas those to PMv originate predominantly from lateral regions (green and grey dots), with small zones of overlap in pre-SMA, the cingulate motor areas, the border between PMd-c and PMv-c, and the most medial part of PMd-r. Figure 7 illustrates more clearly the topography of callosal labelling after three small injections in monkey Mk6.

### Quantitative analysis

A quantitative analysis was conducted on data from 11 injections in 3 monkeys (Mks 1–3) following the procedure described in the Methods section. This procedure provided a numerical estimate of the contribution of each area to the overall callosal afferent connectivity of PMd-r, PMd-c, PMv-r and PMv-c. The results of this analysis are represented graphically for each monkey in Figure 8A (see also [Additional file 1 ]).

Figure 8A shows the variability across the animals with large injections of tracers in the four premotor sectors (Mks 1–3). The variability appeared most prominently for the percentage of the homotopic callosal projections, which ranged from 29 to 40% for PMd-r (in 2 monkeys), from 43 to 81% for PMv-r, from 16 to 23% for PMd-c and from 14 to 19% for PMv-c. For the two latter divisions, the variability was larger for the percentage of the inputs coming from the heterotopic contralateral PMv-r (Fig. 8A). Despite this variability, it appears that PMd-r and PMv-r receive most of their callosal inputs from rostral frontal areas, especially for PMd-r; Inputs from caudal regions are weak or absent. By contrast, PMd-c and, to a lesser extent, PMv-c receive inputs from both rostral and caudal areas.

Figure 8B compares the results of the present study with those previously reported for pre-SMA and SMA-proper [45], using the same analysis of percent contribution of contralateral frontal areas. It appears that the variability observed here is in the same order of magnitude as that observed among 4 monkeys with injections in pre-SMA and SMA-proper.

## Discussion

**Figure 9:**
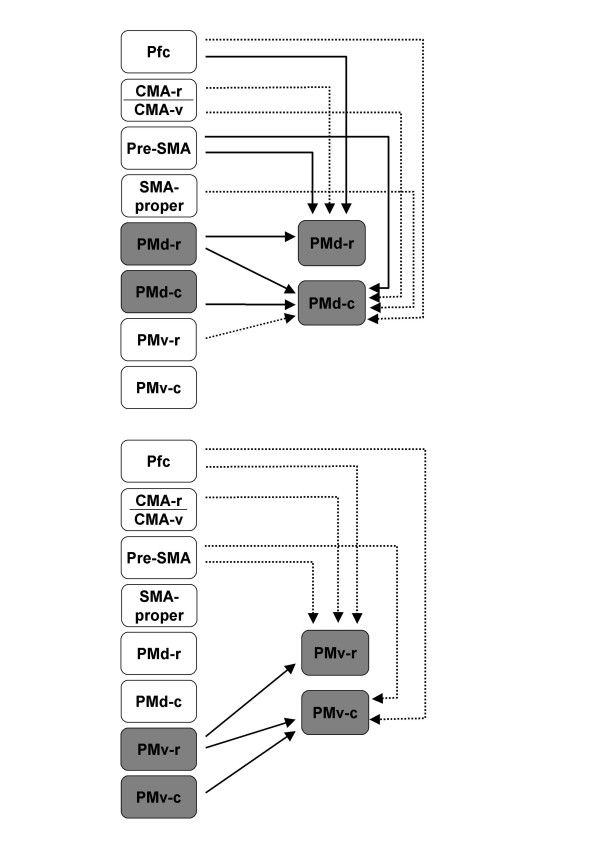
Summary diagram of callosal projections from premotor areas and prefrontal cortex to dorsal and ventral premotor sectors. Thin lines depict strong to moderate projections, dotted lines represent weak projections.

We found that the four premotor areas receive homotopic callosal connections and have distinct patterns of callosal inputs from heterotopic areas. Based on both qualitative and quantitative analyses, the overall results (schematically illustrated in Fig. 9) can be summarized as follows: (1) Callosal inputs to PMd and PMv are organized along a medio-lateral axis; (2) Ventral premotor sectors receive callosal afferents from a limited number of contralateral frontal areas, whereas the dorsal sectors receive inputs from a larger set of areas; (3) The strongest callosal inputs to rostral sectors (PMd-r and PMv-r) were always found to originate from homotopic regions, irrespective of the size of the injection sites and the tracer injected. However, the results following injections into PMv-c and PMd-c varied depending on the size of injections. Small injections yielded preferential labelling in homotopic areas, whereas large injections tended to results in strong labelling in rostral sectors as well; (4) PMd-r stands out as the lateral premotor area with the strongest inputs from the prefrontal cortex, extending from the principal sulcus to cingulate sulcus; (5) caudal sectors (PMd-c and PMv-c) receive weak callosal projections from M1, which does not project to the rostral sectors. We will discuss these findings in relation with previous studies and their functional implication, and address two issues that might affect our interpretations and conclusions, the size of the injection sites and the definition of the borders between areas.

### Relation to previous studies

Ipsilateral connections of premotor and motor cortex gained a tremendous interest in recent years, but callosal connectivity has received less attention, with the exception of M1 and the SMA which were extensively studied. It was found that callosal afferents to M1 and SMA depend on the somatotopic organization, namely with the hand area of M1 receiving much less callosal projections than proximal territories [62]. With the identification of finer subdivisions within the non primary premotor cortex, it is important to examine callosal connectivity of each discrete area in order to advance our understanding of their respective function. Of particular interest is the comparative approach in the same animal, where the spatial distribution of the callosal projecting neurons and their respective contribution to the projection can be directly compared. Two recent studies have adopted such an approach by making injections of two distinct tracers, one in each area, in the same brain. One has compared the callosal afferents of pre-SMA and SMA-proper [63], the other compared those of PMd-r and PMd-c [64]. Both studies reported that each of these premotor areas receives callosal inputs primarily from its counterpart area in the opposite hemisphere and, additionally, from other areas of the frontal cortex.

In the present study, we made comparisons along two axes within the lateral premotor cortex, the rostro-caudal axis (rostral versus caudal regions) and the medio-lateral axis (dorsal versus ventral areas). We found that the general pattern of callosal connectivity described previously holds true, with however some surprising observations which we discuss later in this section. Indeed, we found that the strongest callosal projections to PMd-r and PMv-r arise from their counterpart areas, as was reported for PMd-r [64], pre-SMA and SMA-proper [63], irrespective of the size of the injections. However, unexpectedly this was not systematically the case for PMd-c and PMv-c (see Fig. 8A), which were found to receive their strongest callosal inputs from the rostral sub-regions, i.e. from PMd-r and PMv-r, respectively. This result contrasts with those of Marconi et al. [64] regarding PMd-c, which they reported to receive most of its inputs from its contralateral counterpart. Whether this discrepancy is due to technical differences or the location of the injection sites in the two studies is not clear. One likely cause may be the size of the injections, which were much bigger in our Mks 1–3 than in the 2 monkeys in the study of Marconi et al. [64]. This interpretation is supported by our data in cases with small injections in PMd-c and PMv-c (e.g. Mk6), showing a majority of labelled neurons in the counterpart area on the opposite hemisphere.

As in the case of pre-SMA and SMA-proper [61,63], additional callosal projections to the four subdivisions of PM were found to arise from a number of heterotopic areas. Interestingly, the strength and topography of callosal connections were found to vary along the antero-posterior axis. Our findings indicate that, if all projections are taken into account (see Fig. 9 and supplementary material), caudal areas (PMd-c and PMv-c) receive inputs from a larger set of areas than rostral ones (PMd-r and PMv-r), paralleled by a larger number of projecting cells. A similar result was reported by Marconi et al. [64] for PMd-r and PMd-c. Furthermore, the caudal divisions tend to be connected with caudal premotor areas of the opposite hemisphere including SMA-proper, dorsal and cingulate motor areas (CMA-v, CMA-d). Callosal projections from M1 were rarely observed, and were weak. By contrast, the rostral divisions receive inputs from rostral premotor areas, such as pre-SMA and CMA-r, and from prefrontal cortex but do not receive projections from M1. This general principle was also described for pre-SMA versus SMA-proper [63] and for PMd-r versus PMd-c [64]. In particular, our observations regarding inputs from M1 are compatible with the findings of previous studies showing that contralateral M1 projects weakly to SMA-proper and PMd-c, but does not project to pre-SMA nor PMd-r [61,63,64,74,75]. Likewise, the present findings are in agreement with previous reports regarding the callosal inputs from prefrontal cortex to other premotor areas. Thus, it was found that pre-SMA and PMd-r receive inputs from contralateral prefrontal cortex, but not SMA-proper or PMd-c [63,64].

Finally, callosal projections to each area examined in the current study were found to arise from largely segregated populations of cells, but this segregation was much more striking between cells projecting to dorsal versus ventral sectors (see Figs. 6 and 7), than between rostral versus caudal sectors. In fact, following injections of different tracers, one in either PMd-r or PMd-c the other in PMv-c or PMv-r, large cortical regions contained neurons that were labelled with only one tracer. The zones of co-existence of cells labelled with one or the other tracer were limited to medial premotor areas. Despite this co-existence, a fine examination indicates that the two cell populations were organised in separate patches. The situation is somewhat different for the comparisons between PMd-c and PMd-r on one hand, and between PMv-c and PMv-r on the other hand. Callosal cells projecting to PMd-c and those projecting to PMd-r co-exist within several areas with the strongest overlap in PMd-r, pre-SMA and CMA-r. Cells projecting to PMv-c and those projecting to PMv-r co-exist within PMv-r, CMA-r and to a limited extent in pre-SMA. These findings suggest that dorsal and ventral premotor areas belong to separate inter-hemispheric circuits, but their respective subdivisions belong to partly overlapping anatomical systems.

### Callosal and ipsilateral connectivity of dorsal and ventral premotor areas: a gradient between prefrontal cortex and motor cortex

It is important to examine callosal and ipsilateral connectivity of the lateral premotor areas before speculating on possible functional implications of the present results. Ipsilateral cortical inputs to PMd and PMv have been the focus of recent anatomical studies (see Introduction for references). Despite slight discrepancies between the findings of these studies, there is a general agreement that projections that arise from parietal cortex and prefrontal cortex are organized along the two axes examined in the present study: the rostro-caudal and medio-lateral axes. Along the medio-lateral axis, it was found that parietal and prefrontal areas located dorsally and medially project to PMd-c and PMd-r, those located laterally project to PMv-r and PMv-c. Hence, PMd receives inputs from the dorsal aspect of dorsolateral prefrontal (DLPf) cortex [25,28,37,76] and from the posterior parietal cortex [29,41,45,47,53,54,75,77,78]. By contrast, PMv is connected with the ventral aspect of DLPf cortex and the inferior parietal lobule [12,47,54]. Along the rostro-caudal axis, it was shown, in particular, that areas located more caudally in the superior parietal lobule and the parieto-occipital sulcus project predominantly to rostral PMd, those located more anteriorly project mostly to caudal PMd. Some of these parietal areas that project to PMd-r are directly connected with extrastriate visual cortex and are involved in early visuo-motor transformations [78-80]; those that project to PMd-c are involved in somatosensory processing, and/or sensori-motor transformations [41,42,45,47,54]. On the other hand, PMd-c (but not PMd-r) projects to ipsilateral M1 and to the spinal cord [81,82]. The situation is less clear for PMv-c versus PMv-r in this respect. However, it is interesting to note that the general scheme where ipsilateral and callosal inputs converge remains valid. For example, inputs from the parietal lobe come from the second somatosensory area (S2), among other areas [41,54]. In the present study, we found callosal inputs to PMv-c and PMv-r from S2 (and to a much less extent from S1). Functionally, S2 and PMv may share sensorimotor signals involved in grasping objects [54].

## Conclusion

In summary, lateral premotor areas that receive prefrontal inputs also receive projections from areas involved in early visuo-motor transformations; those that do not receive prefrontal inputs project to M1 and the spinal cord and receive projections from parietal areas involved in high order sensorimotor processing. It is known that rostral and caudal divisions are interconnected, supposedly allowing a functional gradient linking prefrontal cortex with motor cortex. This organization seems to hold true for callosal connectivity. Rostral divisions of lateral premotor cortex, especially PMd, receive callosal inputs from prefrontal cortex, but not from M1, and from their homotopic areas. Caudal divisions, by contrast, do receive inputs from M1, although weak, but have little inputs from prefrontal cortex. Furthermore, PMd-c and PMv-c receive strong callosal inputs from PMd-r and PMv-r, respectively.

Taken together, the anatomical data reviewed above suggest that the general principle of ipsilateral and callosal connectivity of premotor areas remains similar. This seems to argue that callosal connectivity provides similar but complementary information necessary for sensori-motor transformations and bimanual coordination. It is widely accepted that inter-hemispheric connections of motor areas are necessary for the execution of complex motor behavior that requires coordination of both limbs. As reviewed above and elsewhere [e.g. [8,84]], visuomotor information derived from the posterior parietal cortex may reach rostral premotor regions via ipsilateral projections, or indirectly through ipsilateral prefrontal cortex or through callosal fibers (present study; [75,83]). These regions play a key role in high order motor planning, and have projections to caudally adjacent areas, which in turn have direct input to M1 and the spinal cord and their neuronal activity correlates with the kinematics of limb movements. Their callosal connectivity might allow selection of which arm to use, as well as temporal and spatial coordination of bimanual movements. Weak callosal connections of the M1 hand area could reflect the high degree of lateralisation of its neuronal activity during movement execution [84]. By contrast, callosal interactions between premotor areas may convey high order information independent from body representation. In fact, the rostral PMd is involved in spatial attention (see [85] for review) and eye movements [67]. Interestingly, callosal projections to PMd-r sites where eye movements are represented do not differ from those that result from injections at other PMd-r sites. Furthermore, we noted that our injection at an eye movement-related site (Mk8, Table 1; not illustrated) did not lead to any callosal labelling in frontal regions where oculomotor areas would be expected to be located based on sulcal landmarks (e.g., the frontal eye fields on the anterior bank of the arcuate sulcus). This suggests that callosal connections of premotor areas investigated in this study mediate high order information necessary for action planning, independent of the motor effectors.

## Methods

The data reported in this paper are based on 23 tracer injections made in eight macaque monkeys (3 *Macaca fascicularis *and 5 *Macaca mulatta*). Table I summarizes the location of these injections, the nature and amount of the tracers injected. Figure 2 shows their reconstructions on lateral views of the brain. The injections made in monkeys (Mks) 4–8 have been used to determine ipsilateral connections of premotor cortex [54], and those in Mks 1–3 for assessing the degree of overlap/segregation of thalamocortical projections to PM [65]. In addition, BDA injections in cases Mks 1–3 served for studying the corticothalamic projections of PM [66]. Twelve injections were made in PMd (5 in PMd-r and 7 in PMd-c); eleven injections were in PMv (6 in PMv-r and 5 in PMv-c). We used fluorescent tracers Fast Blue (FB), Diamidino-Yellow (DY) and Fluoro-ruby (FR), and the non fluorescent tracers Biotinylated Dextran Amine (BDA) and Choleratoxin B subunit (CB). Experimental procedures have been performed in accordance with the Guide for the Care and Use of Laboratory Animals (ISBN 0-309-05377-3; 1996) and approved by national veterinary authorities (Switzerland and France).

### Surgery

The monkeys (aged 4–10 years and weighing 4–10 kg) were pre-anesthetized with ketamine (5 mg/kg, i.m.) and later deeply anesthetized with propofol (3 ml/kg/h; i.v.). The animals were then placed in a stereotaxic frame. Surgery was performed under aseptic conditions, and body temperature, heart and respiration rates, O_2 _blood saturation and expired CO_2 _were monitored during surgery. The skull was opened on one side in order to expose the premotor cortex and visualize the arcuate and central sulci. In Mks 1–4, injections in the PM subdivisions were guided visually based on sulcal landmarks (arcuate and central sulcus), taking the genu of the arcuate sulcus as the rostrocaudal limit between PMd-r and PMd-c as well as between PMv-r and PMv-c, as described earlier [63,65,67,68]. In Mks 5–8, the locations of the injections were in addition guided using intracortical microstimulation, as described earlier for the same animals [54]. Injections were made with a Hamilton syringe (5 or 10 μl) which was inserted perpendicularly to the cortical surface. At the end of the injections, the dura mater, muscles, and skin were sutured and the animals were treated for several days with analgesics (Vetalgin; 100 mg/kg, i.m. or Rymadil; 4 mg/kg, s.c.), and with an antibiotic (Ampiciline 10%; 30 mg/kg, i.m.). The animals survived for 2–3 weeks and were then deeply anesthetized with ketamine, followed by a lethal dose of sodium pentobarbital (Vetanarcol; 90 mg/kg, i.p.). Transcardiac perfusion with 500 ml of saline (0.9%) was followed by 3 litters of a solution of paraformaldehyde (4% in phosphate buffer 0.1 M, pH 7.6) and 2 litters of a solution of paraformaldehyde (4% in a 10% sucrose solution in phosphate buffer). The perfusion was then continued with 20 and 30% solutions of sucrose in phosphate buffer (2 and 1 litters, respectively). The brain was dissected into blocs, stored during 2–4 days in a solution of 30% sucrose, frozen, and cut in the frontal plane. Sections (50 μm thick) were collected in eight series. Two series of sections were immediately mounted on slides (without cover slip) and stored in the refrigerator for fluorescent microscopy analysis. The histological processing to visualize CB and BDA was described in detail in previous reports [66,69,70]. In Mks 4–8, DY, FB and FR labelled neurons were plotted on sections taken at 0.8 mm intervals, using the MicroBrightField Neurolucida System (Colchester, USA). In Mks 1–3, labelled neurons were plotted using a home made motorized microscope stage, as previously reported [66,69,70]. The DY, FB and FR labelled neurons were plotted on the same sections, whereas the non-fluorescent tracers BDA and CB were each plotted on two sections adjacent to the one analyzed for DY, FB and FR. For each tracer, plots were made every 1.6 mm for reconstruction and illustration purposes, but observation of the labelling was done at 0.8 mm intervals. When necessary, intermediate slides were used for a finer assessment of changes in labelling. Drawings with plots of labelled cells were then exported in the form of computer files formatted for later processing using the software CorelDraw 9. In Mks 1–3, the plots with CB and BDA were aligned and superimposed to the plots with FB and DY, allowing direct comparison of the 4 tracers on the same section (Figs. 5 and 6).

### Definition of areal borders

In Mks 1–3, adjacent sections were processed for SMI-32 (Sternberger Monoclonal Inc., MD, USA), an antibody directed against a non-phosphorylated neurofilament protein labelling pyramidal cells in the cerebral cortex, according to the following protocol. Briefly, free-floating sections were first pre-incubated for 10 min in 1.5% H_2_O_2 _in phosphate-buffered saline (PBS; pH = 7.2) to remove endogenous peroxidase activity. Sections were rinsed several times in PBS, and then incubated overnight at 4°C in SMI-32 monoclonal antibody (dilution 1:3000), 2% normal horse serum and 0.2% triton-X-100. After several rinses, sections were incubated 30–60 minutes at room temperature in biotinylated secondary antibody (1:200, Vector Laboratories, Burlingame, CA) and stained with the avidin-biotin complex (ABC) immunoperoxidase method (Vectastain Elite kits, Vector Laboratories). The reaction was visualized with 3,3'-diaminobenzidine tetrahydrochloride (DAB) as the chromogen, diluted 0.05% in Tris-saline with 0.001% H_2_O_2_. Sections were then washed thoroughly and immediately mounted on gelatin-coated slides, dehydrated, and cover slipped. As a control, the primary antibody was omitted from the processing of some sections while the rest of the procedure remained the same. Another series of sections was stained for Nissl.

SMI-32 immunoreactivity provided reliable criteria to set the limit between PMd-c and PMv-c as shown in previous studies [9,47], and the limit between PM and prefrontal cortex or M1 [63,71]. Photomicrographs illustrating criteria based on SMI-32 were shown in a recent report [63] for the borders PMd-c/PMv-c, PMd-r/Pfc, SMA-proper/PMd-c, pre-SMA/PMd-r, SMA-proper/CMA-d and pre-SMA/CMA-r. Further examples of SMI-32 stained sections are shown in Figure 3 to illustrate the following limits based on SMI-32 immunoreactivity: Pfc/PMv-r, PMd-c/M1, lateral border of PMv-r with the promotor area (ProM, as defined by Paxinos et al. [72]) and the lateral border of PMv-c with the somatosensory cortex. Other borders were based on previously published work. For example, the limit between CMA-d and CMA-v corresponds to the fundus of the cingulate sulcus, based on the distribution of corticospinal neurons [17,73]. Nissl and SMI-32 criteria were also used to define the border between CMA and the region CgG of the cingulate gyrus. Nissl and SMI-32 reconstructions were digitized and aligned to the sections containing the plots of labelled neurons.

### Quantitative analysis

The distribution of labeled neurons was analyzed quantitatively for 11 out of the 23 injections (in Mks 1–3). For each tracer, the labeled neurons were counted on all reconstructed sections. Then, the percentage of labeled neurons in each cortical area was calculated as the ratio of the number of labelled cells in that area to the total number of callosal labelled neurons for a given tracer injection. This procedure provided a numerical estimate of the contribution of each area to the overall callosal afferent connectivity of PMd-r, PMd-c, PMv-r and PMv-c (Fig. 8A; see also supplementary material).

## List of abbreviations used

Ar = arcuate sulcus

BDA = biotinylated dextran amine

C = cerebral cortex

CB = cholera toxin B subunit

CC = corpus callosum

Cd = caudate nucleus

CE = central sulcus

CgG = cingulate gyrus

CIN = cingulate sulcus

Cl = claustrum

CMA-d = dorsal part of the cingulate motor area

CMA-r = rostral part of the cingulate motor area

CMA-v = ventral part of the cingulate motor area

DLPF = dorsolateral prefrontal cortex

DY = diamidino-yellow

FB = fast-blue

FR = fluoro-ruby

GP = globus pallidus

ICMS = intracortical microstimulation

P = sulcus principalis

M1 = primary motor cortex

Pfc = prefrontal cortex

PM = premotor cortex

PMd-c = caudal part of the dorsal premotor cortex

PMd-r = rostral part of the dorsal premotor cortex

PMv-c = caudal part of the ventral premotor cortex

PMv-r = rostral part of the ventral premotor cortex

pre-SMA = rostral part of the SMA

ProM = promotor area

Put = putamen

SMA = supplementary motor area

SMA-proper = caudal part of the SMA

SMI-32 = antibody directed against a nonphosphorylated neurofilament protein that labels pyramidal cells

SomC = somatosensory cortex

S1 = primary somatosensory cortex

S2 = secondary somatosensory cortex

Thal = thalamus

WGA = wheat germ agglutinin

WM = white matter

## Authors' contributions

DB contributed to injections, histological processing, data collection and analysis of monkeys Mk4–8. He also drafted and revised the paper. JTG contributed to injections, histological processing, data collection and analysis in monkeys 4–8. TW contributed to injections, histological processing, data collection and analysis in Mks 1–3. EMR contributed to injections, histological processing, data collection and analysis of all 8 monkeys. He also contributed to drafting and revising the paper.

## Supplementary Material

Additional File 1 Tables 2A, 2B and 2C. Quantitative analysis in Mk1, Mk2 and Mk3 respectively. Click here for file

Additional File 2 Distribution of callosal labelling in monkey Mk3. Click here for file
