# Supplementary material for: Callosal connections of dorsal versus ventral premotor areas in the macaque monkey: a multiple retrograde tracing study
Source: BMC Neurosci. 2005 Nov 25;6:67. doi: 10.1186/1471-2202-6-67 (PMC1314896; doi:10.1186/1471-2202-6-67)
Supplement: Additional File 1 — Tables 2A, 2B and 2C. Quantitative analysis in Mk1, Mk2 and Mk3 respectively. [file 1471-2202-6-67-S1.doc]

Table 2A: Quantitative analysis in Mk1

| *Injeced area (tracer)* | PMd-r (CB) | | PMv-r (BDA) | | PMd-c (FB) | | PMv-c (DY) | |
| --- | --- | --- | --- | --- | --- | --- | --- | --- |
| *Labelling* | n cells | % | n cells | % | n cells | % | n cells | % |
| pre-SMA | 268 | 9 | 2 | 0 | 420 | 22 | 180 | 7 |
| CMA-r | 317 | 11 | 12 | 2 | 152 | 8 | 97 | 4 |
| PMd-r | 851 | 29 | 1 | 0 | 600 | 32 | 27 | 1 |
| PMv-r | 50 | 2 | 349 | 58 | 39 | 2 | 1248 | 49 |
| Pfc | 977 | 34 | 30 | 5 | 26 | 1 | 27 | 1 |
| SMA-proper | 27 | 1 | 0 | 0 | 86 | 5 | 6 | 0 |
| CMA-d | 14 | 0 | 0 | 0 | 64 | 3 | 9 | 0 |
| CMA-v | 73 | 3 | 0 | 0 | 86 | 5 | 12 | 0 |
| PMd-c | 27 | 1 | 0 | 0 | 331 | 18 | 15 | 1 |
| PMv-c | 0 | 0 | 0 | 0 | 3 | 0 | 484 | 19 |
| GgG | 292 | 10 | 0 | 0 | 34 | 2 | 21 | 1 |
| M1 | 0 | 0 | 0 | 0 | 34 | 2 | 5 | 0 |
| Other | 6 | 0 | 209 | 35 | 2 | 0 | 406 | 16 |
| Total | 2902 | 100 | 603 | 100 | 1877 | 100 | 2537 | 100 |

Table 2B: Quantitative analysis in Mk2

| *Injected area (tracer)* | PMd-r (BDA) | | PMv-r (CB) | | PMd-c (DY) | | PMv-c (FB) | |
| --- | --- | --- | --- | --- | --- | --- | --- | --- |
| *Labelling* | n cells | % | N cells | % | n cells | % | n cells | % |
| pre-SMA | 429 | 13 | 155 | 9 | 514 | 11 | 258 | 9 |
| CMA-r | 106 | 3 | 268 | 15 | 247 | 6 | 154 | 6 |
| PMd-r | 1330 | 40 | 68 | 4 | 1804 | 40 | 182 | 7 |
| PMv-r | 60 | 2 | 750 | 43 | 34 | 1 | 741 | 27 |
| Pfc | 1287 | 39 | 267 | 15 | 411 | 9 | 486 | 18 |
| SMA-proper | 4 | 0 | 4 | 0 | 169 | 4 | 15 | 1 |
| CMA-d | 2 | 0 | 3 | 0 | 155 | 3 | 37 | 1 |
| CMA-v | 9 | 0 | 35 | 2 | 221 | 5 | 142 | 5 |
| PMd-c | 16 | 0 | 7 | 0 | 703 | 16 | 20 | 1 |
| PMv-c | 0 | 0 | 13 | 1 | 71 | 2 | 398 | 15 |
| GgG | 73 | 2 | 34 | 2 | 85 | 2 | 48 | 2 |
| M1 | 0 | 0 | 0 | 0 | 60 | 1 | 102 | 4 |
| Other | 1 | 0 | 128 | 7 | 4 | 0 | 153 | 6 |
| Total n cells | 3317 | 100 | 1732 | 100 | 4478 | 100 | 2736 | 100 |

Table 2C: Quantitative analysis in Mk3

| *Injeced area (tracer)* | PMv-r (FB) | | PMd-c (CB) | | PMv-c (BDA) | |
| --- | --- | --- | --- | --- | --- | --- |
| *Labelling* | n cells | % | n cells | % | n cells | % |
| pre-SMA | 42 | 4 | 524 | 18 | 57 | 5 |
| CMA-r | 36 | 3 | 141 | 5 | 7 | 1 |
| PMd-r | 3 | 0 | 416 | 14 | 10 | 1 |
| PMv-r | 955 | 81 | 285 | 10 | 501 | 46 |
| Pfc | 1 | 0 | 108 | 4 | 0 | 0 |
| SMA-proper | 1 | 0 | 244 | 8 | 25 | 2 |
| CMA-d | 0 | 0 | 57 | 2 | 5 | 0 |
| CMA-v | 0 | 0 | 147 | 5 | 1 | 0 |
| PMd-c | 1 | 0 | 684 | 23 | 16 | 1 |
| PMv-c | 8 | 1 | 125 | 4 | 156 | 14 |
| GgG | 28 | 2 | 142 | 5 | 15 | 1 |
| M1 | 0 | 0 | 45 | 2 | 26 | 2 |
| Other | 109 | 9 | 25 | 1 | 269 | 25 |
| Total n cells | 1184 | 100 | 2943 | 100 | 1088 | 100 |
