# Supplementary figures and images for: Callosal connections of dorsal versus ventral premotor areas in the macaque monkey: a multiple retrograde tracing study
Source: BMC Neurosci. 2005 Nov 25;6:67. doi: 10.1186/1471-2202-6-67 (PMC1314896; doi:10.1186/1471-2202-6-67)

## Slide 1
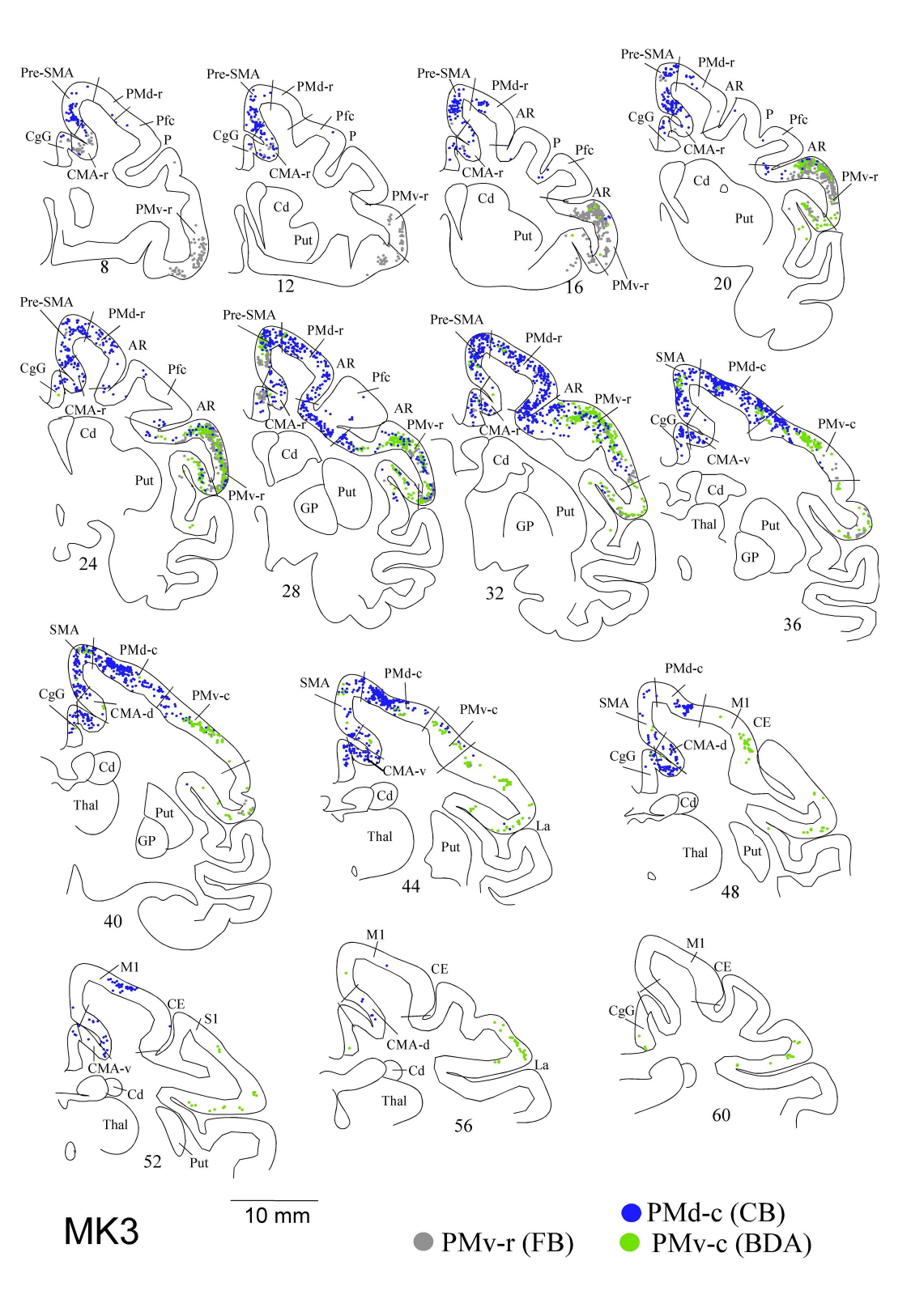

Supplement: Additional File 2 — Distribution of callosal labelling in monkey Mk3. [file 1471-2202-6-67-S2.ppt]
